# Even patients with mild COVID‐19 symptoms after SARS‐CoV‐2 infection show prolonged altered red blood cell morphology and rheological parameters

**DOI:** 10.1111/jcmm.17320

**Published:** 2022-04-13

**Authors:** Marijke Grau, Lars Ibershoff, Jonas Zacher, Janina Bros, Fabian Tomschi, Katharina Felicitas Diebold, Hans‐Georg Predel, Wilhelm Bloch

**Affiliations:** ^1^ 14926 Institute of Cardiovascular Research and Sports Medicine, Molecular and Cellular Sports Medicine German Sport University Cologne Cologne Germany; ^2^ 14926 Department of Preventive and Rehabilitative Sports and Performance Medicine Institute of Cardiovascular Research and Sports Medicine German Sport University Cologne Cologne Germany

**Keywords:** nitric oxide, red blood cell aggregation, red blood cell deformability, red blood cell osmoscan, red blood cells, SARS‐CoV‐2

## Abstract

Infection with the novel severe acute respiratory syndrome coronavirus‐2 (SARS‐CoV‐2) and the associated coronavirus disease‐19 (COVID‐19) might affect red blood cells (RBC); possibly altering oxygen supply. However, investigations of cell morphology and RBC rheological parameters during a mild disease course are lacking and thus, the aim of the study. Fifty individuals with mild COVID‐19 disease process were tested after the acute phase of SARS‐CoV‐2 infection (37males/13 females), and the data were compared to *n* = 42 healthy controls (30 males/12 females). Analysis of venous blood samples, taken at rest, revealed a higher percentage of permanently elongated RBC and membrane extensions in COVID‐19 patients. Haematological parameters and haemoglobin concentration, MCH and MCV in particular, were highly altered in COVID‐19. RBC deformability and deformability under an osmotic gradient were significantly reduced in COVID‐19 patients. Higher RBC‐NOS activation was not capable to at least in part counteract these reductions. Impaired RBC deformability might also be related to morphological changes and/or increased oxidative state. RBC aggregation index remained unaffected. However, higher shear rates were necessary to balance the aggregation‐disaggregation in COVID‐19 patients which might be, among others, related to morphological changes. The data suggest prolonged modifications of the RBC system even during a mild COVID‐19 disease course.

## 1 INTRODUCTION

The severe acute respiratory syndrome coronavirus‐2 (SARS‐CoV‐2) is a novel coronavirus that was first described in 2019 and that has caused a pandemic of acute respiratory disease called the coronavirus disease 2019 (COVID‐19).^1^ SARS‐CoV‐2 infection might cause light to severe transient diseases, including severe acute respiratory syndrome, coagulopathy, vascular and organ damage, microangiopathy and neurological disease phenotypes not solely related to thrombotic events.^2^, ^3^, ^4^ Typical symptoms associated with the infection were described to include breathlessness, cough, fever, ageusia or loss of smell, but infections might also be asymptomatic. Infections with SARS‐CoV‐2 affect all age groups with men and women being equally affected.^5^ Breathlessness and a reduced fitness were reported by most COVID‐19 patients, and it is suggested that this might be related to altered oxygen uptake into the red blood cells (RBC) and/or oxygen binding and/or oxygen release in this disease. These phenomena might be associated to damages of the beta‐chain of the haemoglobin or an increased formation of methaemoglobin, which increases the oxygen affinity of the undamaged haemoglobin.^6^, ^7^ An altered haematological profile including reduced RBC count or shifted RBC distribution width,^8^ but also changes in the RBC morphology, structure and function might occur during the acute phase of the infection and might provide a further explanation for the described symptoms.^9^, ^10^ COVID‐19 is also described to augment RBC rheology.^11^ In particular, RBC deformability was reduced and RBC aggregation parameters were increased, indicating that key determinants of the blood flow in the microcirculation are limited by COVID‐19 following SARS‐CoV‐2 infection.^12^ This might be associated to structural protein damages and membrane lipid remodelling, which might also affect the cytoskeleton that is of major importance for proper RBC deformability.^13^ RBC deformability is crucial for the oxygen supply within the microcirculation and is determined by the surface‐to‐volume ratio, intracellular viscosity, membrane elasticity^14^, ^15^ and nitric oxide (NO) availability.^16^ RBC NO synthase (RBC‐NOS) activity is one source of NO generation within RBC and RBC NO has been linked to S‐nitrosylation of the cytoskeletal spectrins and RBC deformability changes.^17^ Whether the RBC‐NOS signalling pathway is affected by SARS‐CoV‐2 remains unknown. Moreover, whether RBC rheology is altered in a mild course of this disease has yet not been described. Thus, the aim of the study was to investigate morphological changes, RBC rheology, RBC‐NOS activation and marker for oxidative stress in men and women after recovery from COVID‐19 with mild symptomatic in order to further understand the deleterious impact of SARS‐CoV‐2 on the blood system.

## 2 MATERIAL AND METHODS

### 2.1 Study participants and sample processing

A total of *n* = 50 participants (*n* = 37 male; *n* = 13 female) were tested after an average of 60.7 days after positive PCR result on SARS‐CoV‐2 infection. We thus aimed to avoid acute effects of the infection but also post‐COVID‐19 effects which include symptoms that persist longer than or occur after 12 weeks. An additional *n* = 42 healthy controls (*n* = 30 male; *n* = 12 female) were investigated. Age (range) of the study groups were as follows: COVID‐19 male: 24.0 ± 4.4 years (14–30); female: 24.1 ± 5.5 years (16–35); control male: 24.1 ± 5.6 years (17–37); female: 23.8 ± 6.4 years (16–33). SARS‐CoV‐2 infection was verified by PCR and antibody test. With the exception of four asymptomatic participants, all reported a mild course of disease with typical SARS‐CoV‐2 associated symptoms. None were hospitalized or reported other illnesses. None of the participants was vaccinated against COVID‐19 at the time of blood sampling. Fitness level was above average for all participants. The described protocols align with the Declaration of Helsinki, and all participants gave written informed consent to participate in this study. The project was approved by the local ethics committee of the German Sport University (087/2020).

Venous blood samples were collected at rest from the vena mediana cubiti into EDTA vacutainer (Becton Dickinson GmbH) and further processed immediately. Described parameters were analysed of all tested participants unless otherwise stated.

### 2.2 Measurement technique

Basal RBC parameters including RBC count, haemoglobin concentration (hb), haematocrit (hct), mean corpuscular volume (MCV), mean corpuscular haemoglobin (MCH), mean corpuscular haemoglobin concentration (MCHC) and RBC distribution width (RDW) were directly determined in whole blood using the hematology analyzer Sysmex Digitana KX‐21N (Sysmex).

Red blood cells were separated by centrifugation (3600 *g*, 5 min, room temperature), dispersed on a glass slide, heat fixed and Pappenheim staining was conducted to better illustrate and analyse morphological changes of the RBC. Images of the stained slides were taken using a Zeiss microscope coupled to a CCD‐camera (DXC‐1850P, Sony). Magnification of the images was 200‐fold. A total of five images were taken of each slide, all RBC (average 500 cells per image) were counted and morphological abnormalities were put in proportion as a percentage.

Red blood cells deformability was measured by ektacytometry using the LORRCA MaxSis (RR Mechatronics)^18^ after dilution of 100*10^6^/µl RBC in polyvinylpyrrolidone (PVP) solution (1:250; 29cP at 37°C, RR Mechatronics). The samples were sheared in a Couette system and exposed to nine consecutive shear stresses between 0.3 and 30 Pa, and the diffraction pattern of a laser beam directed through the samples was analysed by the LORRCA software of each applied shear stress resulting in a respective elongation index (EI). The LORRCA software then calculated the maximum elongation index (EImax), representing the maximum deformability at infinite shear stress and SS1/2 which represents the shear stress at one‐half EImax. Finally, the SS1/2:EImax ratio was calculated and higher values display reduced RBC deformability.

The osmotic gradient ektacytometry (osmoscan) was performed using the LORRCA MaxSis and measured deformability under various osmotic conditions. Measured RBC number was standardized for each sample using the following equation: 1000/RBC count = x µl sample mixed with 5 ml PVP. Thus, the deformation of RBC shape at a defined shear stress and constant temperature (37°C) is measured during an osmotic gradient and the following parameters were provided by the LORRCA software: Omin which corresponds to the osmolality at which RBC deformability reaches its minimum in the hypotonic environment and which is influenced by the mean cellular surface‐to‐volume ratio. Below this point, most RBC rupture if the osmolality is further decreased. EImax corresponds to the maximum deformability near the isotonic osmolality and relates to the RBC membrane surface. Ohyper, the osmolality in the hypertonic region, corresponds to 50% of EImax and reflects the hydration status of the RBC. Ohyper is affected by the cytoplasmic viscosity and the cell volume.^19^, ^20^, ^21^

Red blood cells aggregation was measured at 37°C by syllectometry using the LORRCA MaxSis after the hct of the samples was adjusted to 40% using autologous plasma. All samples were fully oxygenated for 15 min with the use of a Roller Mixer (Karl Hecht KG) prior to the measurement.^22^ Oxygenated samples were transferred to the Couette system and changes of backscattered light were recorded over 120 s using two photodiodes and presented as a graph (syllectogram) to calculate an Aggregation‐Index (AI%). The threshold shear rate balancing RBC aggregation and disaggregation was obtained after an iteration procedure was performed to primarily calculate dIsc min. This parameter defines the minimum change in backscatter intensity during the iteration procedure, representing the minimum shear rate where RBC aggregates start to disaggregate (y at dIsc min (s^−1^)).

Nitrotyrosine and phosphorylation of the RBC‐NOS serine 1177 residue were measured by immunohistochemistry.^17^ Thus, nitrotyrosine represents a marker for the generation of free radicals, NO in particular, and a marker for oxidative stress. Phosphorylation of the RBC‐NOS serine 1177 residue has been described as activation site, thus representing increased enzyme activity and hence NO production.^16^, ^17^ Briefly, separated RBC were immediately fixed in 4% formaldehyde, separated on a slide and heat fixed. A test and a control area were marked on each slide, both areas were washed with tris‐buffered saline (0.1 mol TBS, pH 7.6) and incubated with 0.1% trypsin solution. Non‐specific antibody binding was minimized by incubating both areas with 3% skim milk. The test area of each slide was incubated with the respective primary antibody in a 0.3% skim milk solution: Anti‐phospho eNOS (Ser1177; (dilution: 1:150, Millipore), Anti‐Nitrotyrosine (dilution 1:500, Upstate/Millipore). Slides were washed with TBS, treated with 3% normal goat serum (Dako) and incubated with a secondary goat anti‐rabbit antibody (dilution: 1:400, Dako). 3,3‐diaminobenzidine‐tetrahydrochloride (DAB) solution (Sigma) in TBS was used to develop the staining. Slides were dehydrated by exposure to alcohol solutions of increasing concentration and sealed using Entellan^®^ (Merck). Images were taken from the test and the control area of each slide using a Zeiss microscope coupled to a CCD‐camera (DXC‐1850P, Sony). The grey values of the images were analysed using the ‘Image J’ software (National Institutes of Health). A total of 50 RBC from at least four images were determined in the test area and a total of 10 RBC from at least two images were determined in the control area. The grey values were cleared against the background grey value. Finally, grey values of RBC from test and control areas were subtracted to obtain net staining intensities.

Statistical analyses of the data were performed using GraphPadPrism software 8.0. Data were analysed for normal distribution of the data, and one‐way ANOVA was performed to detect effects between the tested groups. Differences were considered as significant with * *p* < 0.05, ** *p* < 0.01 and *** *p* < 0.001. Presented data are mean ± standard deviation (SD).

## 3 RESULTS

### 3.1 Blood parameters

Comparison of RBC parameters indicated reduced MCV, hct, hb and MCH in male COVID‐19 patients compared with healthy male controls. Female COVID‐19 patients showed lower MCV and higher MCHC than the respective control participants. In general, RBC count, hct, hb, MCH and MCHC were lower in females compared to males (Table 1).

**TABLE 1:** Red blood cell parameters of male and female COVID‐19 participants compared with respective female and male healthy control group

	Male Control	Male COVID−19	Female Control	Female COVID−19
RBC [*10^6^/µl]	4.8 (0.5)	4.7 (0.5)	4.5 (0.4)#	4.4 (0.3)+++
Hct [%]	43.7 (4.2)	41.4 (4.4)*	40.5 (3.3)##	38.9 (3.1)+++
Hb [g/dl]	15.1 (1.4)	14.2 (1.8)*	13.4 (0.9)###	13.1 (1.4)+
MCV [fl]	90.5 (3.5)	87.2 (4.2)***	91.0 (3.7)	88.3 (3.3)*
MCH [pg]	31.1 (1.5)	29.9 (2.3)**	30.2 (1.4)#	29.8 (2.0)
MCHC [g/dl]	34.4 (1.6)	34.4 (1.5)	33.2 (1.1)#	33.5 (1.7)*
RDW [%]	12.7 (0.7)	13.1 (1.6)	12.8 (0.7)	12.7 (0.8)

Data are mean (SD).

* *p* < 0.05; ** *p* < 0.01; *** *p* < 0.001 vs respective control; # *p* < 0.05; ## *p* < 0.01 female vs male control; + *p* < 0.05 female vs male COVID‐19.

### 3.2 Morphological changes

**FIGURE 1:**
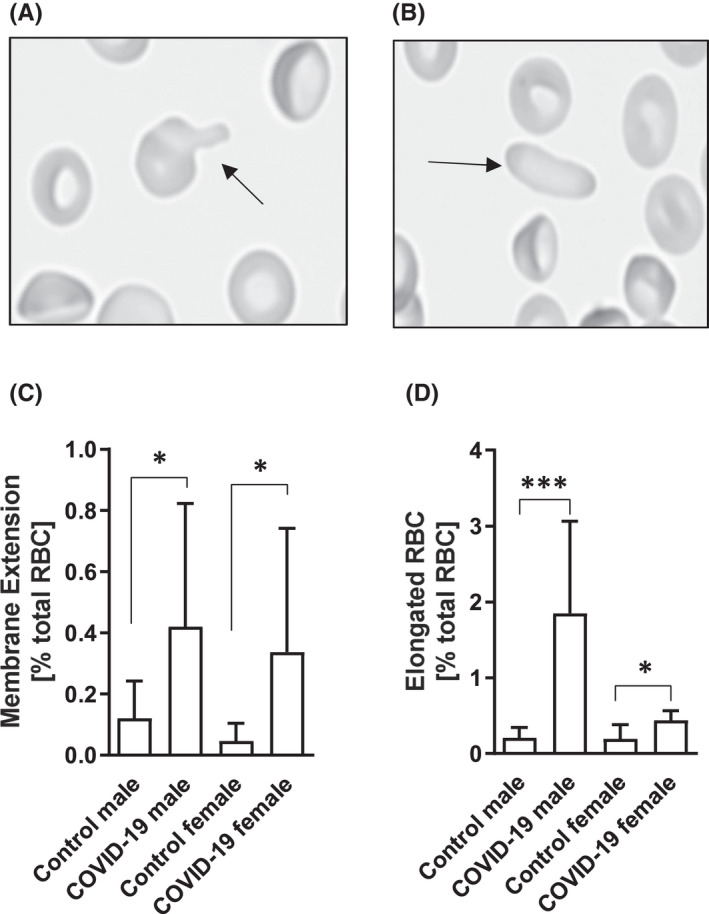
Morphological changes of RBC in COVID‐19 patients. Representative images of morphological changes including (A) membrane extensions and (B) permanent elongated RBC. Images were taken with 200‐fold magnification. Quantitative analyses of an average of 2500 RBC revealed (C) a significantly higher percentage of RBC showing membrane extensions after SARS‐CoV‐2 infection in both, female and male, groups compared with the respective control groups (* *p* < 0.05). (D) In parallel, percentage of permanent elongated RBC was also significantly higher in the COVID‐19 groups with higher values in the affected male cohort (*** *p* < 0.001) compared to the female cohort (* *p* < 0.05). Data are mean ± SD

Distinct morphological changes, especially alterations of the membrane, were observed in samples from COVID‐19 patients. Two major changes were identified and analysed of *n* = 20 (11 m/9 f) COVID‐19 patients and *n* = 15 (8 m/7 f) controls. Membrane extensions were defined as evaginations of the membrane leading to an apex which can be observed at the RBC (Figure 1A). These changes were significantly higher after SARS‐CoV‐2 infection in both, males and females compared with healthy controls (Figure 1C). Permanent elongated RBC (Figure 1B) were also significantly higher in both COVID‐19 groups compared to the respective control groups (Figure 1D).

### 3.3 Red blood cell deformability

**FIGURE 2:**
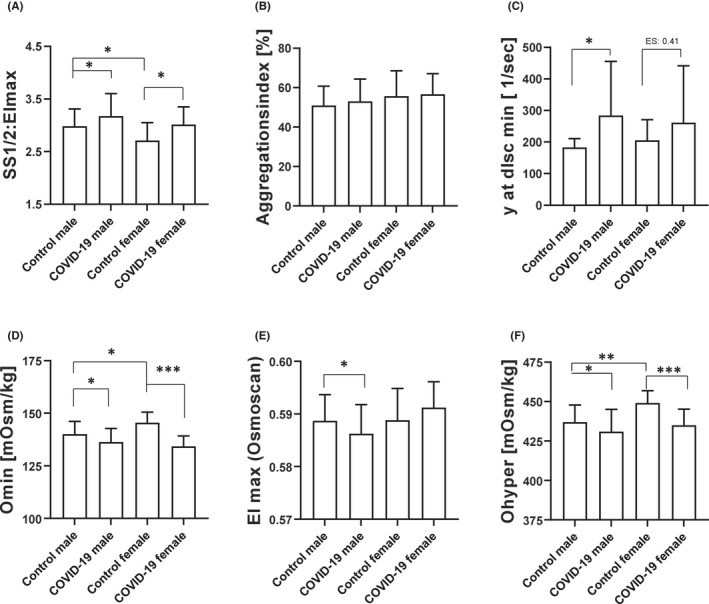
Influence of COVID‐19 on RBC rheological parameters. (A) RBC deformability was displayed by SS1/2: EImax ratio and values were significantly higher, and thus RBC deformability was significantly lower, in both COVID‐19 groups (* *p* < 0.05, respectively). Comparison of the control groups revealed higher RBC deformability in females compared with males (* *p* < 0.05). (B) Aggregation index was comparable between the tested groups. (C) Shear rate balancing RBC aggregation and disaggregation was significantly higher in males (* *p* < 0.05) after SARS‐CoV‐2 infection and a similar trend was observed in females, although not statistically significant. Osmoscan parameters revealed (D) lower Omin in both COVID‐19 groups (* *p* < 0.05 and *** *p* < 0.001, respectively), and also lower Omin values in females compared with males (* *p* < 0.05). (E) EImax values measured during the osmoscan showed lower values in males after SARS‐CoV‐2 infection (* *p* < 0.05) but no difference between the female cohorts or between male and female controls. (F) Ohyper was significantly lower in male and female COVID‐19 groups compared to the respective controls (* *p* < 0.05 and *** *p* < 0.001). Control females showed significantly higher values compared with male controls (** *p* < 0.01). Data are mean ± SD

Red blood cell deformability was represented as SS1/2 to EImax ratio. SS1/2:EImax values were significantly higher and thus, RBC deformability was significantly lower in both COVID‐19 groups. Comparison of male and female controls revealed higher RBC deformability in females compared to males (Figure 2A).

### 3.4 Red blood cell aggregation

Red blood cell aggregation was analysed from *n* = 48 (30 m/18 f) COVID‐19 patients and *n* = 25 (15 m/10 f) controls. Values of RBC aggregation index (AI%) did not differ between control and COVID‐19 (Figure 2B). The threshold shear rate balancing RBC aggregation and disaggregation was significantly higher in male COVID‐19 compared with the respective control group. In the female test group, values tend to be higher compared with the control cohort (effect size: 0.41; Figure 2C).

### 3.5 Red blood cell Osmoscan

Omin was reduced after SARS‐CoV‐2 infection in both, female and male COVID‐19 cohorts (Figure 2D). EImax (osmoscan) was significantly reduced in males but not in females with COVID‐19 (Figure 2E) and Ohyper was also significantly reduced in both COVID‐19 groups compared with their respective control groups. Comparison of male and female controls showed higher Ohyper values in females than in males (Figure 2F).

### 3.6 Phosphorylated RBC‐NOS serine 1177

**FIGURE 3:**
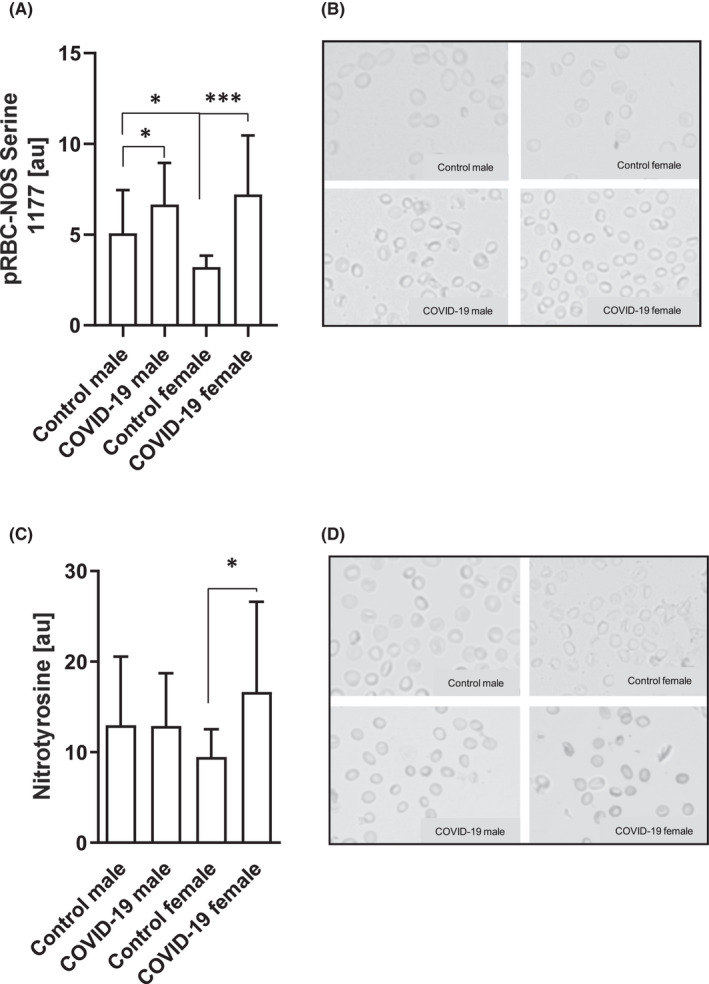
Altered RBC‐NOS activation and nitrotyrosine in COVID‐19 patients. (A) Phosphorylation of the RBC‐NOS serine 1177 residue, and thus, the activation of the enzyme, was significantly higher in both COVID‐19 groups (* *p* < 0.05 and *** *p* < 0.001). Comparison of the control cohorts revealed higher RBC‐NOS serine 1177 phosphorylation in males compared with females (* *p* < 0.05). (B) Representative images of phosphorylated RBC‐NOS serine 1177 staining. Magnification: 200‐fold. (C) Nitrotyrosine levels were significantly higher in COVID‐19 females (* *p* < 0.05) while no such difference was observed in the male groups. (D) Representative images of nitrotyrosine signal of the study groups. Magnification: 200‐fold. Data are mean ± SD

Higher RBC‐NOS serine 1177 phosphorylation is related to an increase in enzyme activation.^17^, ^23^ Values were analysed from *n* = 33 (20 m/13 f) COVID‐19 patients and *n* = 20 (11 m/9 f) controls. Increased staining intensity of phosphorylated RBC‐NOS serine 1177 residue was detected in both, males and females with COVID‐19 compared with respective healthy controls. Control females showed lower RBC‐NOS serine 1177 phosphorylation compared with control males (Figure 3A,B).

### 3.7 Nitrotyrosine

Nitrotyrosine staining was performed in *n* = 33 (23 m/1 0f) COVID‐19 patients and *n* = 20 (10 m/10 f) controls. Intensity of nitrotyrosine staining was similar between male controls and male COVID‐19 patients. In contrast, females showed higher staining values in COVID‐19 compared with healthy controls (Figure 3C,D).

## 4 DISCUSSION

Severe SARS‐CoV‐2 dependent COVID‐19 disease was shown to affect the RBC system^24^, ^25^ and RBC rheological parameters in particular.^12^ This might be related to morphological changes^9^ and might impact oxygen transport and supply. The recent study aimed to address the question whether a mild COVID‐19 disease progress is related to prolonged alterations in RBC morphology and rheological parameters.

The data were separated by gender because the results confirm recent findings of distinct differences in the haematological profile between males and females in general^26^, ^27^ which was associated to higher testosterone levels in males^28^ or periodic menstrual blood losses of females.^29^ Distinct differences between males and females were also observed in the rheological parameters showing higher deformability in healthy females compared with healthy males which might be explained by regular menstruation and thus, a potentially younger circulating RBC population which were described to have higher deformability compared to older RBC.^26^, ^29^, ^30^

Studies on the effects of SARS‐CoV‐2 infection suggest that the extent of altered haematological parameters were associated to the severity of the COVID‐19 disease and have been described to predict the clinical outcome of the patients.^31^, ^32^, ^33^ A drop in haemoglobin is well documented for severe and critically ill COVID‐19 patients.^34^, ^35^, ^36^ SARS‐CoV‐2 interacts with the haemoglobin molecules within RBC possibly resulting in haemoglobin denaturation, drop in functioning haemoglobin, decreased NO availability and oxidative stress.^34^ It is also discussed that modifications of the haemoglobin might affect oxygen uptake, binding and release.^6^ Analysis of the results from the mild COVID‐19 cases investigated herein revealed reduced haemoglobin concentration and mean cellular haemoglobin in COVID‐19 patients, thus confirming previous findings^33^ and further suggesting that even patients with a mild disease state and overcome infection experience a high impact of the virus on circulating blood cells. In addition, haematocrit and MCV values significantly decreased in COVID‐19. Both the parameters are interrelated because the haematocrit concentration is calculated by MCV multiplied by the number of circulating RBC which was comparable between COVID‐19 and the respective control groups. Thus, while the number of circulating RBC remained constant in mild COVID‐19 but the MCV decreased, the hct also decreased. Changes in MCV might be related to alterations of the cell morphology. RBC of COVID‐19 patients, both males and females, showed distinct morphological changes similar to those previously described for patients with severe COVID‐19 illness.^9^, ^10^ The extent of the defined elongated RBC and membrane extensions was lower compared with seriously ill patients. Data presented herein indicate that about 2.5% of RBC from male COVID‐19 patients showed membrane defects, which was a higher percentage than observed in females with about 1% of RBC defects. This means that 200–500 billion circulating cells show membrane defects given the fact that about 20 trillion RBC circulate through the human body. Because blood sampling described herein was scheduled approximately 60 days after infection to exclude the acute infection phase, it seems plausible that the amount of RBC defects was most likely higher during acute infection state. It seem reasonable that these structural changes limit RBC deformation which is one reason for erythrophagocytosis by macrophages^37^ and which might indicate accelerated RBC turnover possibly explaining anaemia^35^ during COVID‐19 infection. Indeed, RBC deformability was significantly lower in both COVID‐19 groups underlining previous findings.^11^, ^12^ Moreover, deformability under various osmotic conditions showed significant differences between COVID‐19 and healthy participants. EImax (osmoscan), Omin and Ohyper were significantly lower in male and female COVID‐19 patients respectively. Thus, COVID‐19 dependent changes of the RBC shifted the osmsocan curve to the left and downwards as described for stiffened cells^19^ concluding that the described haematological and morphological changes impairs RBC deformability even after recovery from mild COVID‐19 disease. Since RBC deformability is crucial for the passage of the small capillaries and oxygen supply within the microcirculation, it is suspected that stiffening of RBC, next to haemoglobin alterations, might also be associated to the described hypoxemia^38^ during COVID‐19 infection. RBC aggregation represents another crucial rheological parameter which was described to be affected by acute severe COVID‐19 disease.^12^, ^39^ The present data could not support these findings, possibly because of the mild nature of the disease course reported herein or because the enrolled subjects did not show additional diseases which were described for the COVID‐19 patients by Renoux and colleagues.^12^ Though, shear rate balancing aggregation and disaggregation was remarkably higher in the COVID‐19 groups and it seems plausible that the membrane evaginations and elongation of the cells described earlier increase the contact area between adjacent RBC thus increasing forces connecting these cells.

Further, nitrotyrosine levels were measured to investigate a possible role of oxidative stress in the observed functional changes. Nitrotyrosine originates from the reaction of NO and superoxide resulting in peroxynitrite which in turn nitrates tyrosine residues of proteins. Thus, nitrotyrosine represents a marker for cellular damage and oxidative stress.^40^ Elevated levels of RBC free radical content have been previously described to induce a reorganization of the membrane structure as well as water and ion imbalance^41^ which was also linked to reduced RBC deformability.^42^, ^43^, ^44^ Damage of RBC proteins and membrane lipid remodelling in COVID‐19 patients was reported by Thomas et al.^13^ The observed changes include fragmentation of crucial RBC cytoskeleton proteins such as spectrin which is essential for RBC deformability,^17^ but also of the anion exchanger 1/band 3 essential for membrane stability.^45^ The authors concluded that modifications of the RBC proteins by COVID‐19 might explain the lack of the cells to respond to environmental oxygen saturation and/or oxidative stress.^13^ In the present study, nitrotyrosine levels were increased in female COVID‐19 patients confirming, at least in part, higher free radical content in RBC of COVID‐19 patients which promotes the decline in RBC deformability. Such a relation was not observed in male COVID‐19 patients. However, since a large number of different free radical species were described to be present in RBC, further studies including the investigation of other free radical species need to be carried out. High free radical content was also described to reduce activation of the NO producing enzyme RBC‐NOS.^42^ RBC‐NOS produced NO has been linked to S‐nitrosylation of RBC cytoskeletal proteins including spectrin, thus positively affecting RBC deformability^17^ and possibly preserving deformability in conditions of oxidative stress.^46^ Surprisingly, RBC‐NOS activation, represented by the phosphorylation state of the serine 1177 residue,^16^ was significantly higher in both COVID‐19 groups. Thus, also higher NO levels might be hypothesized in RBC of COVID‐19 patients because higher RBC‐NOS activation was associated to increased levels of RBC NO.^16^, ^17^, ^47^ In addition, the generation of nitrotyrosine requires the presence of NO as described above. A study by Mortaz and colleagues report increased NO levels in RBC of severe COVID‐19 patients^48^ which might apply to the finding of the present study. Given the reduced RBC deformability in COVID‐19 patients despite higher RBC‐NOS activation, it is assumed that this observation might represent a compensatory mechanism, which, however, cannot maintain RBC deformability.^49^, ^50^ It seems plausible that the observed morphological changes were caused by damages of the cytoskeleton, the membrane or both and that increased RBC‐NOS produced NO is not capable to affect deformability because of the structural damages.

In conclusion, to the best of our knowledge, these are the first data revealing prolonged prominent RBC structural and rheological changes in patients after a mild COVID‐19 disease. These changes were observed in both, young female and young male COVID‐19 patients although certain haematological parameters and morphological changes seemed to be more pronounced in male patients. Impairment of RBC deformability and aggregate strength, morphological changes and oxidative stress seem to be highly interrelated. Impaired rheological parameters were shown to affect blood flow dynamics and, together with the reported left‐shift of the oxygen dissociation curve,^6^ possibly oxygen supply in the microcirculation. Investigations of the RBC system post‐COVID‐19 (>12 weeks after infection) but also investigations on the exact underlying mechanisms of altered RBC structure and rheological parameters are needed to develop specific therapies and/ or therapeutic agents.

## CONFLICT OF INTEREST

The authors declare no conflict of interest.

## AUTHOR CONTRIBUTIONS

**Marijke Grau:** Data curation (lead); Formal analysis (lead); Investigation (equal); Methodology (lead); Resources (equal); Supervision (equal); Visualization (lead); Writing – original draft (lead). **Lars Ibershoff:** Formal analysis (equal); Investigation (equal); Writing – review & editing (equal). **Jonas Zacher:** Conceptualization (lead); Funding acquisition (lead); Project administration (lead); Resources (equal); Writing – review & editing (equal). **Janina Bros:** Formal analysis (equal); Investigation (equal); Writing – review & editing (equal). **Fabian Tomschi:** Formal analysis (equal); Investigation (equal); Writing – review & editing (equal). **Katharina Felicitas Diebold:** Investigation (equal); Project administration (equal); Writing – review & editing (equal). **Hans‐Georg Predel:** Conceptualization (equal); Funding acquisition (equal); Project administration (equal); Resources (equal); Writing – review & editing (equal). **Wilhelm Bloch:** Conceptualization (equal); Project administration (equal); Resources (equal); Writing – review & editing (equal).

## PATIENT CONSENT

All patients gave written informed consent to participate in this study.

## Data Availability

The data that support the findings of this study are available on reasonable request from the corresponding author. The data are not publicly available due to privacy or ethical restrictions.
